# Predation on sentinel prey increases with increasing latitude in *Brassica*‐dominated agroecosystems

**DOI:** 10.1002/ece3.9086

**Published:** 2022-07-11

**Authors:** Hannah L. Gray, Juliano R. Farias, Madelaine Venzon, Jorge Braz Torres, Lucas Machado Souza, Rafael Carlesso Aita, David A. Andow

**Affiliations:** ^1^ Department of Entomology University of Minnesota‐Twin Cities Minneapolis Minnesota USA; ^2^ Universidade Regional Integrada do Alto Uruguai e das Missões Santo Ângelo Brazil; ^3^ Empresa de Pesquisa Agropecuária de Minas Gerais ‐ EPAMIG Viçosa Brazil; ^4^ Departamento de Agronomia‐Entomologia Universidade Federal Rural de Pernambuco Recife Brazil; ^5^ EMBRAPA‐Recursos Genéticos e Biotecnologia Brasília Brazil

**Keywords:** agroecology, aridity, arthropods, biogeography, *Brassica oleracea*, community ecology, ecosystem services

## Abstract

In natural ecosystems, arthropod predation on herbivore prey is higher at lower latitudes, mirroring the latitudinal diversity gradient observed across many taxa. This pattern has not been systematically examined in human‐dominated ecosystems, where frequent disturbances can shift the identity and abundance of local predators, altering predation rates from those observed in natural ecosystems. We investigated how latitude, biogeographical, and local ecological factors influenced arthropod predation in *Brassica oleracea*‐dominated agroecosystems in 55 plots spread among 5 sites in the United States and 4 sites in Brazil, spanning at least 15° latitude in each country. In both the United States and Brazil, arthropod predator attacks on sentinel model caterpillar prey were highest at the highest latitude studied and declined at lower latitudes. The rate of increased arthropod attacks per degree latitude was higher in the United States and the overall gradient was shifted poleward as compared to Brazil. PiecewiseSEM analysis revealed that aridity mediates the effect of latitude on arthropod predation and largely explains the differences in the intensity of the latitudinal gradient between study countries. Neither predator richness, predator density, nor predator resource availability predicted variation in predator attack rates. Only greater non‐crop plant density drove greater predation rates, though this effect was weaker than the effect of aridity. We conclude that climatic factors rather than ecological community structure shape latitudinal arthropod predation patterns and that high levels of aridity in agroecosystems may dampen the ability of arthropod predators to provide herbivore control services as compared to natural ecosystems.

## Graphical abstract

Arthropod predation on herbivore prey is higher at lower latitudes in natural ecosystems, yet it remains unknown whether this pattern persists in human‐dominated ecosystems, where frequent disturbances often alter local predator community structure. Here, we examined predation on model caterpillars and community structure in a human‐dominated organic Brassica oleracea agricultural ecosystem in the United States and Brazil. In both countries, we found a reversed predation gradient, whereby arthropod predation was higher at higher latitudes, however, the change in predation with latitude likely due to distinct continental patterns of aridity.

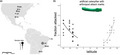

## 1 INTRODUCTION

In the 19th century, Alfred Russell Wallace first proposed that biotic interactions between many plants and animal taxa may be stronger at lower latitudes, creating a latitudinal biotic interaction gradient parallel to the latitudinal biodiversity gradient (Wallace, 1878). Researchers in the 21st century continue to examine this Latitudinal Biotic Interaction Hypothesis (LBIH), both to understand current patterns in biodiversity and biotic interactions, and to anticipate the effects of climate change on future global macroecological patterns (Bale et al., 2002; Schemske et al., 2009). Recently, a meta‐analysis examined the accumulated empirical evidence on the LBIH and found that both herbivory and predation decrease significantly with increasing latitude and that this pattern was strongest for ectothermic predators (Zvereva & Kozlov, 2021). One potential explanation for this biogeographic pattern is Paine's Predator Hypothesis, which states that at lower latitudes, predator communities are more diverse, and that diversity creates redundancies and complementation in prey consumption that drives equatorial increases in predation (Paine, 1966; Pianka, 1966). However, Zvereva and Kozlov (2021) suggest that climate may be the critical factor driving changes in predation intensity observed in ectothermic predators. Local and regional studies have explored how predator community composition and climate influence predation, yet little is known about how both factors may contribute to latitudinal predation gradients, particularly in human‐dominated ecosystems.

At least 45% of global land cover is dominated by human activity including areas used for housing, transportation, and food production (Hooke et al., 2012). Unlike most natural ecosystems, human‐dominated ecosystems experience frequent and diverse disturbances, such as tillage, planting, and weed management actions known to perturb ectothermic arthropod predators (Schowalter, 2012). These disturbances make agroecosystems an ideal setting in which to test the extent of latitudinal predation gradients and the proposed underlying mechanisms. If predator diversity drives higher predation rates at lower latitudes, then the latitudinal predation gradient may be much weaker in agroecosystems due to the reduced plant and arthropod diversity characteristic of most food production systems (Gonthier et al., 2014). Alternatively, the homogenization of both global crop taxa and crop‐associated cosmopolitan arthropod predators and herbivores may give rise to similar predation rates regardless of location, resulting in no detectable gradient (Bebber et al., 2014). Still, if the climate is the major factor driving latitudinal predation gradients, then arthropod predation gradients could exist with similar strength in agroecosystems as previously documented in primarily forest ecosystems (Jeanne, 1979; Novotny, 2006; Roslin et al., 2017), given the large regional scale at which climate acts.

Arthropod predators provide crucial herbivore pest control services in agroecosystems. Therefore, considerable attention has been directed toward understanding the ecological conditions associated with stronger predation rates. Regional studies reveal that predation on herbivore prey usually increases with greater arthropod predator densities and diversity, both of which are supported by the provisioning of key predator resources through plant diversity management (Gurr et al., 2017; Root, 1973). Plant diversity within and near crop fields is determined by a combination of cultivar selection and tolerance for non‐crop plants. When tolerated in‐field, weed plants may contribute significantly to overall plant richness and could follow the latitudinal plant diversity gradient found in natural ecosystems (Barthlott et al., 2007). The inclusion of diverse weed, crop, and wild plants within and near crop fields often increases the availability of shelter, nectar, alternative prey, and pollen resources required by many arthropod predators to persist and reproduce (González‐Chang et al., 2019). However, the effect of predator resource availability on predator diversity, density, and attack rates appears to depend greatly on the specific arthropod and plant taxa present (Andow, 1991; Schellhorn & Sork, 1997). It is unclear whether the taxa associated with globally replicated cropping systems change with latitude in ways that impact the effect of resource availability on predation intensity.

Climate may influence latitudinal predation patterns indirectly through shifts in arthropod predator resource availability and directly through effects on predator behavior, altering mortality to predators and predator lifespans. Within a given ecosystem, arthropod predator attacks on sentinel prey have been found to peak during wet seasons when preferred prey are most abundant, suggesting an indirect influence of climatic conditions on predation through prey resource availability (Molleman et al., 2016). More directly, an experimental warming experiment showed that increased temperatures can elicit increased interference and competition among co‐occurring predators such that predation rates decrease (Barton & Schmitz, 2018). Yet, a globally distributed experiment revealed that higher temperatures at lower latitudes are correlated with increased predation rates (Romero et al., 2018). The incorporation of community data into such globally distributed experiments could illuminate whether the effects of climate on predation are due to shifts in resources, species distributions, or behavior.

As emphasized by Roslin et al. (2017), standardized methods are crucial to detecting large‐scale biogeographic patterns in predation. To evaluate the reach and drivers of latitudinal arthropod predation patterns we assessed arthropod predator attack rates on uniform sentinel caterpillar baits in organic agroecosystems dominated by a single crop species, *Brassica oleracea* (L.). The *B. oleracea* system is well‐researched as a globally grown staple vegetable crop and hosts a cosmopolitan pest‐herbivore community, including *Brassica*‐specialist caterpillars upon which we modeled prey baits (Grzywacz et al., 2010). By surveying arthropod and plant communities and measuring predation intensity within *Brassica* study sites located across 15° latitude in the United States and 21° latitude in Brazil we assessed Paine's (1966) Predator Hypothesis which predicts that predator attack rates increase with higher predator richness at lower latitudes, against the hypothesis that climate factors mediate the effect of latitude on predation through direct effects on predator communities and indirect effects on predator resources.

## 2 MATERIALS AND METHODS

### 2.1 Site selection

We assessed predation rates in *B. oleracea*‐dominated agroecosystems across latitudinal transects in the central United States (30°–45°N) and eastern Brazil (8°–30°S) during the 2017 growing season (Table S1). We selected 5 study sites in the United States and 4 study sites in Brazil. In each site, study plots were embedded in similar background native ecosystems (grasslands and savannas) with access to a metropolitan area market to minimize between‐plot and site variation in growing practices. All plots were located on practicing organic farms that eschewed applications of insecticides that could interfere with arthropod predator function (Bommarco et al., 2011). Variation in farmer weed management and inter‐cropping practices created a range of within‐field vegetational diversity among plots within each site. On the high end of vegetation, diversity was plots planted in polyculture cropping schemes with low weeding intensity whereas plots on the low end of this spectrum practiced monoculture cropping and intensively weeded to remove most additional plant diversity. Elevation averaged 273 (SE = 14 m) meters in United States plots and 766 (SE = 84) meters among Brazilian plots (Table S1). At each site, we established study plots in production fields containing predominantly leafy kale or collard cultivars in the mid‐ to late vegetative growth stage, with each plot measuring approximately 20 × 20 m. Fields planted in flowering varieties of *B. oleracea* including broccoli and cauliflower were occasionally used, and the open leaf structure of these cultivars closely aligned with the architecture of leafy varieties. In total, we assessed predation rates in 55 *Brassica* sites with an average of 6.11 (sd = 2.67) distinct plots per site in nine sites (Table S1). This compares favorably with previous studies, which assessed predation at two (Jeanne, 1979), three (Novotny, 2006), or five (Roslin et al., 2017) sampling plots per site with five to eight sites total.

### 2.2 Predation assessment methods

We followed established methods to hand‐roll 0.5 × 3 cm artificial caterpillars, designed to mimic late‐instar *Trichoplusia ni* (L.) larvae, an important *Brassica* pest, from green plasticine (Van Aken Plastalina 10508) (Howe et al., 2009; Roslin et al., 2017; Sam et al., 2015). At each study site, an average of 18.25 (sd = 3.33) caterpillar baits were glued with Loctite™ Control Superglue to the underside of the central vein of a lower leaf, a common location for late‐instar lepidopteran larvae on *Brassica* crops (Kumar & Omkar, 2018). Baits were exposed for 2 days, as in Roslin et al. (2017), collected into 2 ml centrifuge tubes, and stored on ice until scoring to avoid any non‐predatory indentations in the malleable plasticine material. We used the scoring key developed by Low et al. (2014) to score caterpillars as not‐attacked or attacked by a mammal, bird, or arthropod predators (Figure S1). Missing caterpillar baits (*n* = 31, 3.1%) were removed from the analysis as we could not confirm that the absence was due to predation.

### 2.3 Local vegetation assessment

The same day we deployed the artificial caterpillars, we also surveyed the plant and arthropod communities in each plot. We used the point‐quadrat method (Goodall, 1952) with 80 pins per plot arranged in two perpendicular 20 m rows of 40 vertical pins every half meter, to characterize the plant community. Given the distinct row structure and frequent weed removal in agricultural production, we oriented transects diagonally across rows to capture between and along row variation in vegetation and chose the half‐meter point interval based on the large size of the focal crop (diameter approximately 0.5–1 m) and sparsely vegetated space between rows (1–1.5 m) and to minimize potential damage to crop through point‐dropping (Caratti, 2006). For each point dropped along the transect we recorded if the point was intercepted with bare ground or a plant and identified the plant to species. Plant community data were missing for two of our plots in the Minas Gerias (MG) site and were thus omitted from any analysis using plant data. We then calculated the density of *B. oleracea* and non‐*Brassica* plants as a number of points identified as *B. oleracea* or a non‐*Brassica* plant divided by the number of points observed per plot and species richness for all plants including *B. oleracea* (minimum plant richness = 1).

### 2.4 Local arthropod assessment

To characterize the arthropod community, we randomly selected 12 non‐adjacent *Brassica* plants within our study plot, avoiding plants bordering the edge of the plot, and systematically visually identified and counted arthropods from the foliage crown to stem base, turning over all leaves top to bottom and within a 0.5 m radius around the focal plant. In one site, the Federal District, 24 instead of 12 plants were sampled per plot, therefore we corrected for this oversampling by rarifying arthropod community count data to a random sample of 12 plants per plot for this site prior to analysis. We simplified ant counts to the presence/absence on a plant to account for recruitment behavior and expressed this as the proportion of plants with ants. All arthropods observed on the 12 focal plants were identified in the field to family or species when possible and densities for each taxon were recorded per site. We then characterized taxa by the trophic guild (predator, parasitoid, herbivore, pollinator, and detritivore) and calculated the density and family richness of all arthropod taxa present, as well as for herbivore prey and predator groups. Previous analyses compared results with all predators and those known to specifically attack caterpillars, however, results were not significantly different as most predator taxa observed to consume caterpillars.

### 2.5 Statistical analyses

We performed all statistical analyses using R (R Version 4.1.1, R Core Team, 2018). To examine the relationship between predation and biogeographic factors we used generalized linear mixed‐effects models (GLMM) for proportions, modeling predation as the ratio of the number of baits attacked versus not attacked to assess daily predation risk for each study plot. Assuming binomial distributed errors and a logit‐link function, we modeled predation response as a function of country, latitude (absolute value), and the interaction of country and latitude, adding site nested within the country as a random factor to partition local variation among sampling plots within a site from variation among sites at different latitudes. We included country as a fixed effect in this model due to the lack of latitudinal range overlap between study countries, as Zvereva and Kozlov (2021) found that the strength of the latitudinal predation gradient changed distinctly among distinct climates associated with different latitudinal ranges. Initially, we constructed this model for each type of predator attacking the model caterpillars (arthropod, bird, and mammal) as well as a model for all predator attack types. However, when attacks by a given type of predator occurred at one or fewer sites within a country, data from that country was removed from the model and the model was simplified to include only latitude as a fixed effect and site as a random effect. We fit all predation models with the “glmer” function using adaptive Gauss‐Hermite quadrature in the *lme4* package in R (Bates et al., 2015; Bolker et al., 2009). We assessed model fit by examining plots of residuals versus fitted values and Pearson's goodness of fit statistic and significance of predictors with Wald *χ* ^*2*^ type 3 test of main effects in R. To aid in model fit interpretation we used the “r.squaredGLMM” function in the *MuMIN* package to calculate R^2^_GLMM_ marginal and conditional values using methods developed by (Nakagawa et al., 2017) (Bartón, 2010).

Next, we examined factors known to influence predation that could not be adequately controlled for by site selection that may influence observed latitudinal patterns; namely within‐site variation in vegetational diversity and variation in elevation and climate among sites within a country. Agronomic practices of weed removal, intercropping, and cover cropping are often regionally influenced (Shennan, 2008), therefore we assessed whether vegetational diversity among plots within a given site would obscure latitudinal differences in predation rates by modeling all predation responses as a function of the site, plant richness, and the interaction of site and plant richness, adding country as a random effect. We assessed model fit and fixed effect significance with the same methods as the latitude models above. To characterize climate gradients in our study we collected data from the WorldClim database (Fick & Hijmans, 2017) with the “getData” function in the package *raster* (Hijmans, 2021). Temperature, measured as mean annual temperature (MAT) correlated strongly with latitude among our study sites (Pearson's χ^2^ = 0.90, *p* < .001), therefore was not analyzed as a potential confounding climate factor. Instead, we examined the Köppen aridity index (Aridity Index = Mean Annual Precipitation/[MAT +33], lower index values indicate greater aridity) as this index is a good proxy for plant productivity across a gradient of humid and arid climates in North and South America and was not significantly correlated with the latitudes studied (Pearson's *χ* ^2^ = 0.13, *p* = .341), (Quan et al., 2013). To examine if variation in elevation and aridity could obscure latitudinal patterns in predation we modeled predation response as a function of elevation, aridity, latitude, and country, scaling all continuous variables, allowing for all fixed effect interactions, and adding site nested within country as a random factor. To assess the significance of fixed effect terms we used the function “drop1” in the *lme4* package to sequentially simplify the model, starting with the most complex interactions and computing a likelihood ratio test (LRT) statistic at each step (Hertzog et al., 2017). When removal of a given fixed effect did not significantly impact model fit as indicated by LRT statistic, that term was removed. We assessed the overall model fit as above and constructed new univariate GLMMs with variables in significant interactions to estimate unscaled impacts on predation rates.

**FIGURE 1:**
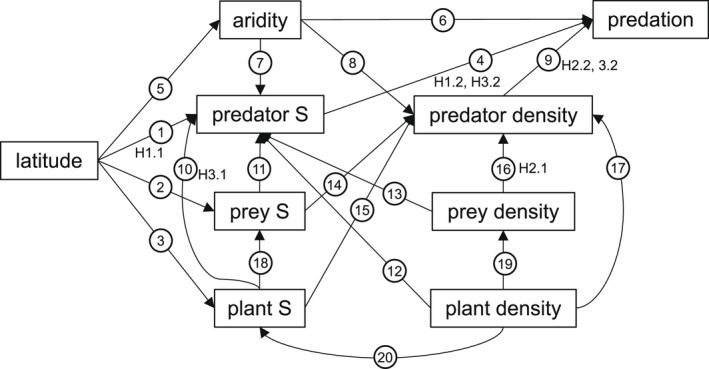
A priori models tested with piecewise structural equation modeling (SEM). Our causal structure incorporates biogeographic and ecological theories to explore the direct and indirect effects of latitude on arthropod predation. In our model, latitude can influence arthropod predation indirectly through changes in predator richness associated with the latitudinal diversity gradient (arrows 1–4) or through changes in aridity (arrows 4–9). Relationships between predators and the resources of predators can modify these indirect latitudinal effects. Predation can be driven by predator richness (arrow 4) or density (arrow 9). Predators require diverse and abundant prey and plant resources to persist in agroecosystems (arrows 10–17) and plant density can, directly and indirectly, influence prey communities (arrows 18–20). Here plant density refers to the density of non‐*Brassica* plants. Richness variables are indicated with the abbreviation S and are measured at the family level for predator and prey richness and the species level for plant richness

To examine whether local biotic and climate effects mediate the effect of latitude on arthropod predator attack rates we conducted piecewise structural equation modeling (SEM) using the *piecewiseSEM* package in R (Lefcheck, 2016). Piecewise SEM can test multiple hypotheses in a single causal network and can incorporate a variety of model structures, distributions, and assumptions (Lefcheck, 2016). We constructed our causal network based on Paine's Predation Hypothesis, ecological theory, and results from our biogeographic pattern analysis. We tested the following predictions related to arthropod predation rate as depicted in Figure 1:

1. Latitude drives the diversity of all taxa, including predators (H1.1) and predation is stronger where predators are more diverse (H1.2) (Paine, 1966).
2. Predator density is dependent on prey density (H2.1) and predation pressure increases with greater densities of predators (H2.2) (Wangersky, 1978).
3. More diverse cropping systems contain a greater amount of diverse shelter, nectar, alternative prey, and pollen resources that support more abundant and diverse predator communities (H3.1), which enhances arthropod predation rates (H3.2) (Root, 1973).
4. Aridity, shown to mediate the effect of latitude on predation in our biogeographic pattern analysis, influences the composition of arthropod predator communities (Tsafack et al., 2019).

Prior to analysis, we rescaled all continuous predictor variables to zero mean and unit variance using the “decostand” function in the *vegan* package (Oksanen et al., 2020) so that our parameter estimates reflect effect size among variables measured at very different scales (Nieminen et al., 2013). We then used the *car* package (Fox et al., 2021) to run a variation inflation factor (VIF) analysis with all predictor variables to check models for variable independence (Zuur & Ieno, 2016). We found no variables to have a VIF score greater than the conservative threshold of 3, thus all variables are included in our causal network. The seven component models examined (1) the effect of aridity, predator richness, and predator density on arthropod predation rates, (2) the effect of latitude on aridity, (3) the effect of aridity, latitude, non‐*Brassica* plant density and richness, and prey density and richness on predator density and (4) richness, (5) the effect of latitude and non‐*Brassica* plant richness on prey richness, (6) the effect of non‐*Brassica* plant density on prey density, and (7) the effect of non‐*Brassica* plant density and latitude on non‐*Brassica* plant richness. All component models were fit as GLMMs with site nested within the country as a random factor. We examined tests of directed separation to identify missing significant correlations in our model and updated the component models to include any identified missing interactions, and then evaluated the goodness of fit with Fisher's C Test (Sudnick et al., 2021). For each endogenous variable, we examined both the marginal and conditional *R* ^2^ values.

## 3 RESULTS

### 3.1 Attacks on model caterpillars

The mean recovery rate per site of model caterpillars was 96.9 ± 7% (Table S1). Of the 974 recovered model caterpillars, we detected 276 instances of attack, the majority of which was attributable to arthropods (90.2%). Birds accounted for the second most attacks (6.1%), followed by mammals (3.2%). However, model caterpillars were only attacked by birds and reptiles in one plot each in Brazil, and by mammals in one plot in the United States (Table S1, Figure S1). Attack rates on model caterpillars by arthropods were not significantly different between the two study countries (LRT *χ* ^*2*^ = 0.13, *p* = .71).

### 3.2 Effect of latitude on predation

**FIGURE 2:**
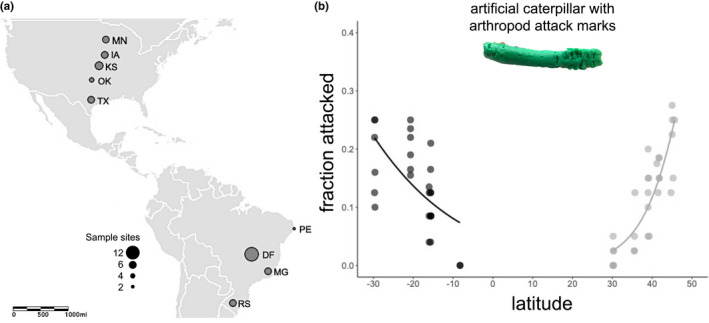
(a) Map of study sites marked by their state abbreviations and with a number of sampled field plots indicated by dot size and (b) scatter plot showing arthropod predation rate measured as the daily fraction of model caterpillars attacked by arthropod predators by latitude for each study plot in Brazil (black) and USA (gray) *Brassica oleracea* agroecosystems. Size of the site marker on the map scales to the number of sample sites per state. Data points are partially transparent and appear darker when overlapping. Curves are the fitted equations from logistic regression

The prediction of Wallace (1878), and other ecological theoreticians (Paine et al., 1966; Pianka, 1966), did not hold in our agricultural ecosystem. Contrary to previous expectations and studies, we found that daily arthropod predator attacks on model caterpillars increased significantly with increasing latitude and that the strength of this pattern varied by study country (Wald *χ* ^2^ = 8.65, *p* = .003) (Figure 2b, Table S2). Our model of the effects of latitude and country, controlling for sample site explained 75% of the variance in arthropod predation (Table S2). In the United States, arthropod predation increased significantly faster with latitude than in Brazil (Table S2). For every 1° latitude increase in the United States, the odds of arthropod attack increased by 44.4% (41.5–47.5%, 95% CI) from the odds of 0.03 at the lowest latitude studied (30.1°N; Austin, Texas). In Brazil, the odds of arthropod attack increased more slowly, by 39.4% per 1° latitude (37.8–41.1%, 95% CI) reaching the odds of 0.37 at the highest absolute latitude studied in Brazil (29.7° S; Santa Maria, Rio Grande do Sul). Moreover, the latitude of minimum predation differed by country, with less than 5% baits attacked at 30.1°N in the United States and in Brazil at 8.3°S. We found a similar trend in the effect of latitude on bird predator attacks in the United States, with the odds of bird attacks increasing 43.6% (36.2%–52.5% CI) per degree latitude increase (Wald χ^2^ = 3.19, *p* = .074, Table S2). In contrast, Brazilian mammal predator attacks did not vary with latitude (Wald *χ* ^2^ = 0.19, *p* = .665, Table S2).

### 3.3 Vegetation diversity, elevation, and aridity

**FIGURE 3:**
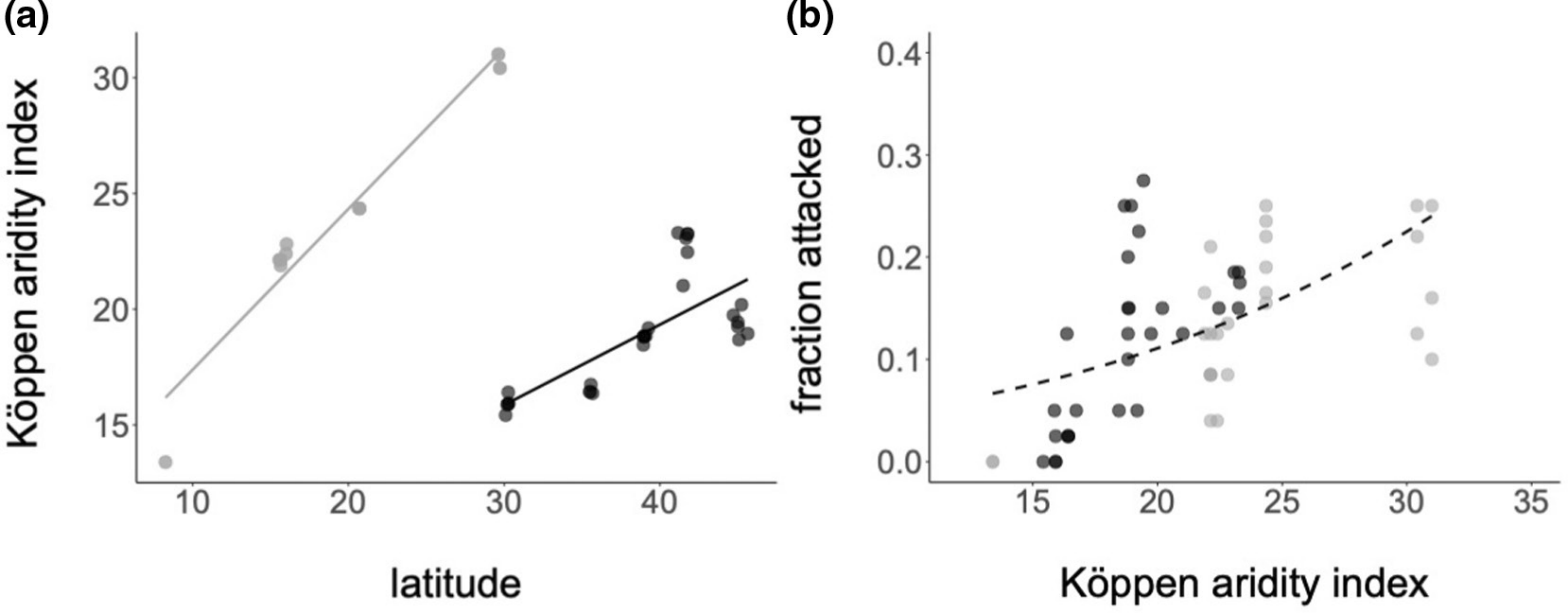
Scatter plots showing (a) Köppen aridity index (higher values = less arid) by latitude and (b) the predation rate (proportion of attacked model caterpillars) by Köppen aridity index for each study site in Brazil (black) and United States (gray) *Brassica oleracea* agroecosystems. Data points are partially transparent and appear darker when overlapping. The curve dashed trend line in panel B is fitted from the logistic regression equation

Neither variation in local site vegetation richness (Tables S3 and S4) nor elevation (Table S5) influenced predation patterns. However, patterns of aridity were associated with the observed predation gradients and may explain the equatorial shift in predation in Brazil (Tables S5 and S6, Figure 3). Our simplified selected model of potential mediating biogeographic factors included all 2‐way interactions of country, latitude, and aridity except the interaction of latitude and country, and explained more variation in arthropod predation as the model of latitude alone (R^2^_GLMM_ = 0.90, Table S5). The model showed a significant interaction of aridity and country (Wald *χ* ^2^ = 12.98, *p* < .001) and a trending interaction of aridity and latitude (Wald *χ* ^2^ = 3.32, *p* = .068) influenced the odds of daily arthropod predator attack (Table S7). Because of differences in continental aridity gradients between study countries, the study site at approx. 30°N was twice as arid, as the study site at approx. 30°S (Figure 3a). In the model to estimate unscaled effects of aridity on arthropod predation, the interaction of country and aridity was not significant (Wald *χ* ^2^ = 0.53, *p* = .467), and for a unit increase in Köppen aridity index (higher values = less arid), odds of arthropod attack increased by 42.7% (37.5–48.6%, 95% CI) (Figure 2b, Table S6).

### 3.4 Direct and indirect effects on arthropod predation (piecewiseSEM)

**FIGURE 4:**
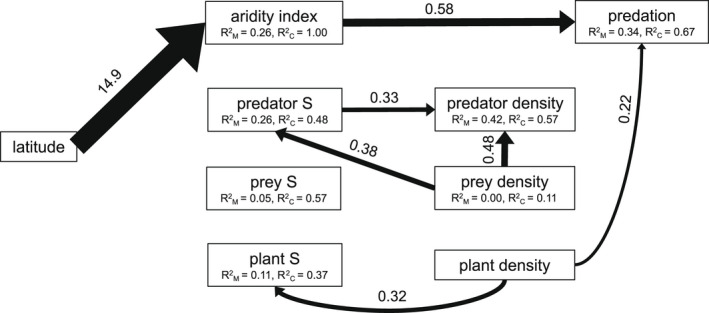
Piecewise structural equation model showing significant (*p* < .05) direct and indirect effects of latitude and local community structure on arthropod predation over all sampled plots (*n* = 55) controlling for the site (*n* = 9) nested within the country (*n* = 2) as a random effect. Arrow width represents relative standardized effect sizes of significant causal paths (see Table S9 for a detailed model summary with non‐significant effects). Plant density refers to non‐focal crop species (*Brassica oleracea*) plant community. Higher values of the Köppen aridity index used in models indicate a less arid climate. Marginal (R^2^_M_) and conditional (R^2^_C_) are given for each component model

The final piecewiseSEM model incorporating aridity, plant (Table S3), predator (Table S7), and prey (Table S8) community variables explained 67% of the variation in arthropod predation rate (AIC = 123.16, Fisher's C = 25.16, *df* = 24, *p* = .397, Table S9). Reinforcing results from GLMM models, piecewiseSEM revealed that aridity (higher AI values) decreased with latitude (standard estimate: *β* = 1.49, *p* = .001) and predation rates increased with lower aridity (*β* = 0.58, *p* = .022) (Figure 4, Table S9). Despite this effect of aridity on predator function, aridity did not influence the density (*β* = 0.13, *p* = .391) or richness (*β* = −0.02, *p* = .995) of arthropod predators. Most predator taxa observed in the field are known to attack caterpillar prey (Table S7). Yet, neither predator density (*β* = 0.10, *p* = .344) nor predator richness (*β* = −0.02, *p* = .857) influenced observed predation rates as expected by the theories that informed our model construction (Figures 1 and 4). However, plots with greater prey densities had greater predator richness (*β* = 0.38, *p* = .002) and predator densities (*β* = 0.48, *p* = .001). Predator densities were also enhanced in plots with greater predator richness (*β* = 0.33, *p* = .045). Though not included in our original model, the test of direct separation detected a missing significant relationship between non‐*Brassica* plant density and arthropod predation that was included in the final model (Figure 4). Arthropod predation was higher in plots with greater non‐*Brassica* plant density (*β* = 0.22, *p* = .028). Latitude did not influence predator, prey, or plant richness in our study system and prey richness was neither a significant predictor nor response of any other variable in our model (Figure 4, Table S9).

## 4 DISCUSSION

Utilizing a standardized agroecosystem and assessment protocol, this study disentangled the influences of local ecological factors and geographic context on latitudinal arthropod predation patterns. In both the United States and Brazil, we found an overall increase in daily arthropod attack rates on sentinel prey with latitude in *Brassica*‐dominated agroecosystems, a reversal of the gradient observed in natural ecosystems (Roslin et al., 2017; Zvereva & Kozlov, 2021). The range of the fraction of sentinel prey attacked daily (0–0.28) was commensurate with previous studies utilizing artificial caterpillar baits across a range of latitudes in natural ecosystems (Roslin et al., 2017), and in agroecosystems studied at a single latitude (Ferrante et al., 2017; Howe et al., 2015). Supporting the hypothesis that climate drives latitudinal patterns in arthropod predation, our results show that the strength of the latitudinal arthropod predation pattern varied significantly between study countries with distinct climates. Specifically, we found that continental patterns in aridity rather than local differences in arthropod predator richness or density mediate the relationship between latitude and predation.

Our site selection methods which emphasized uniformity of background native ecosystems (grasslands and savannas) and production methods (organic) precluded controlling for latitudinal variation in aridity among sites. In central North America, the highest aridity is associated with the desert belt at 27–33°N latitude, which includes our lowest latitude site in central Texas (30°N). The South American aridity gradient in Brazil is not associated with the southern desert belt and is instead related to the climate of the South Atlantic, with the aridest location at our lowest latitude site in Pernambuco (8°S) (Franchito et al., 2014; Silva & Souza, 2018). For both historical and economic reasons, Brazilian population density and agricultural production remain highly concentrated along the Atlantic coast, therefore it was not possible to select study sites with a range in aridity that more closely mirrored sites in the United States (Dias et al., 2016). A twofold difference in aridity between study sites at approximately 30° latitude in each country likely explains the stark differences in the observed predation rates between those two sites and the overall poleward shift in the predation gradient in the United States (Figure 3a).

If temperature alone mediated the effect of latitude on predation, we would have seen a consistent effect of latitude in both countries due to the tight correlation between latitude and mean annual temperature across the agricultural sites studied. A recent survey in China found that carabid predator communities were less stable in drier climates due to an increase in the dominance of single species (Tsafack et al., 2019). It is unclear if similar subtle shifts in species dominance occurred in the *Brassica* agroecosystem studied here, as field constraints limited identification to the family level. Further, all studied plots were irrigated to promote crop growth, therefore how aridity influences predator plant and herbivore prey resources are also unresolved by this study. Our piecewiseSEM model did not detect any missing links between aridity and measures of plant, prey, or predator variables, indicating that this climate variable may be acting independently of community composition on predator function. Under drier climates, arthropod predators may change behaviors to avoid water loss or may benefit more from alternative shelters found outside of the cropping areas such as leaf rolls (Romero et al., 2022). Future studies that prioritize site selection based on contrasts in aridity could better determine if the effect of aridity on arthropod predation is due to shifts in community composition or predator behavior.

Our results do not support Paine's Predator Hypothesis, specifically, that the latitudinal predation gradient arises due to higher predator richness at lower latitudes following the latitudinal diversity gradient. However, little data exists to contextualize why arthropod predator richness in our model agroecosystem was not influenced by latitude. To the best of our knowledge, only three other studies (Jaffe et al., 2007; Jeanne, 1979; Privet & Pétillon, 2020) have applied standardized sampling protocols to assess if arthropod predators are more diverse at tropical latitudes. Results from these studies do support the latitudinal diversity gradient, however, each assessed just one predatory taxon (ants or spiders) and only Jeanne (1979) examined a true latitudinal gradient across more than two sites. Without knowledge of how entire predator assemblages change with latitude in natural ecosystems, we can only conjecture that a failure to meet the first assumption of the predator hypothesis could be attributable to local ecological factors driven by management practices. While we found within‐site variation in weed tolerance and intercropping practices thought to support arthropod predator richness, we did not find variation in these practices among sites. This indicates that the availability of predator resources thought to enhance predator richness (Root, 1973) is independent of latitude in *Brassica* cropping systems.

That neither higher predator richness nor density enhanced arthropod attacks on sentinel prey contradicted our predictions based on ecological theories (Paine, 1966; Root, 1973; Wangersky, 1978). In a community context, arthropod predators exhibit a range of interactions including niche complementation, redundancy, and intraguild predation, with resultant positive, neutral, or negative effects of predator richness and density on predation rates, respectively (Casula et al., 2006). The two most abundant predator groups found in our study system, coccinellid beetles and spiders are known to engage frequently in complex intraguild and extraguild interactions (Hodge, 1999; Finke & Denno, 2002). Further, in response to environmental stressors such as heat or plant community disturbances both coccinellids (Snyder, 2009) and spiders (Schmitz & Barton, 2014) change foraging patterns in ways that may impact predation rates. Future experiments comparing the behavior of globally distributed arthropod predators, such as the coccinellid *Harmonia axyridis* (Pallas) (Roy et al., 2016) across a range of latitudes could clarify if variation in intraguild interactions within predator assemblages in agroecosystems decouples global predation patterns from predicted effects of predator richness and density.

While global patterns of crop losses have not been well documented (Bebber et al., 2014), our finding that predation rates decline with latitude aligns with indirect evidence that herbivory is likely higher in lower latitude agroecosystems. Research on soybean agroecosystems in the United States showed that pesticide application rates decreased by 66% from Louisiana (30°N) to Minnesota (45°N), a similar range of latitudes to those studied here (Deutsch et al., 2018; Ziska, 2014). Much of the focus in temperate agroecosystems has been placed on modifying local and landscape management methods to enhance predation as an ecosystem service (Chaplin‐Kramer et al., 2011; Lichtenberg et al., 2017). However, our results demonstrate that arthropod predation can be highly variable across geographic ranges and is most strongly predicted by aridity rather than management factors. While plots with more area covered in non‐crop plants were associated with greater attack rates this effect was less strong than that of aridity. This suggests that management recommendations aimed at enhancing arthropod predation in agricultural systems likely have variable effectiveness between regions with distinct climates.

The main strength of conducting our study of the Latitudinal Biotic Interaction Hypothesis in a standardized agroecosystem was the ability to examine the contribution of local ecological and climatic factors on predation gradients. Our results suggest that aridity, rather than predator and predator resource characteristics, modulates the effect of latitude on arthropod predation in agroecosystems. This supports previous work that highlights the sensitivity of biotic interactions involving ectothermic predators to changes in climate (Romero et al., 2022; Zvereva & Kozlov, 2021). However, we conclude that arthropod predation increases with latitude in *Brassica* agroecosystems, the opposite pattern observed in natural ecosystems. This distinct biogeographic pattern highlights the need to examine how climate influences key biotic interactions in human‐dominated ecosystems to manage and maintain arthropod‐mediated ecosystem services.

## AUTHOR CONTRIBUTIONS

**Hannah L. Gray:** Conceptualization (lead); data curation (lead); formal analysis (equal); funding acquisition (lead); investigation (lead); methodology (equal); project administration (equal); resources (equal); visualization (lead); writing – original draft (lead); writing – review and editing (equal). **Juliano Farias:** Investigation (equal); resources (equal); writing – review and editing (equal). **Madelaine Venzon:** Investigation (equal); resources (equal); writing – review and editing (equal). **Jorge Torres:** Investigation (equal); resources (equal); writing – review and editing (equal). **Lucas Machado Souza:** Investigation (equal); writing – review and editing (equal). **Rafael Carlesso Aita:** Investigation (equal); writing – review and editing (equal). **David A Andow:** Conceptualization (equal); formal analysis (equal); funding acquisition (supporting); investigation (equal); methodology (equal); project administration (equal); resources (equal); supervision (lead); writing – review and editing (equal).

## CONFLICT OF INTEREST

The authors declare no conflicting interests.

## STATEMENT OF INCLUSION

Our study brought together both researchers and farmer‐stakeholders across the two study countries. Researchers in our study countries were involved in the study design, execution, and interpretation of results. Where relevant literature published by a scientist from Brazil was cited and an effort was made to consider research in Portuguese. In both countries, we met with farmer‐stakeholders prior to research activities and supplied reports for each farmer within 3 months of data collection on their property. Wherever possible we sought to demonstrate our field methods as the model caterpillar predation assessment method readily provides tangible evidence of pest control benefits to farmer‐stakeholders.

## Supporting information


Appendix S1

## Data Availability

All datasets presented herein are available at https://doi.org/10.5061/dryad.73n5tb30h
