# Supplementary material for: Predation on sentinel prey increases with increasing latitude in Brassica‐dominated agroecosystems
Source: Ecol Evol. 2022 Jul 11;12(7):e9086. doi: 10.1002/ece3.9086 (PMC9272068; doi:10.1002/ece3.9086)
Supplement: Supplementary file 1 — Appendix S1 [file ECE3-12-e9086-s001.docx]

**Supplementary Table S1.** Summary of artificial caterpillar monitoring methods, recovery rates, and attacks by predator type for each study site. State-site abbreviations are as follows: Rio Grande do Sul (RS), Minas Gerais (MG), Federal District (DF), Pernambuco (PE), Texas (TX), Oklahoma (OK), Kansas (KS), Iowa (IA), and Minnesota (MN)

|  |  |  |  |  |  |  |  |  |  | **count of bait attacks** | | | | |
| --- | --- | --- | --- | --- | --- | --- | --- | --- | --- | --- | --- | --- | --- | --- |
| **country** | **site** | **plot** | **lat** | **lon** | **elevation (MASL)** | **start** | **end** | **baits (n)** | **recovery (%)** | **arthropod** | **bird** | **lizard** | **mammal** | **all** |
| **Brazil** | **RS** | 1 | -29.72 | -53.72 | 96 | 12-Jun | 14-Jun | 20 | 90 | 8 | 0 | 0 | 0 | 8 |
|  |  | 2 | -29.72 | -53.72 | 96 | 12-Jun | 14-Jun | 20 | 100 | 5 | 0 | 0 | 0 | 5 |
|  |  | 3 | -29.72 | -53.72 | 96 | 12-Jun | 14-Jun | 20 | 100 | 10 | 0 | 0 | 0 | 10 |
|  |  | 4 | -29.62 | -53.69 | 102 | 13-Jun | 15-Jun | 20 | 95 | 6 | 0 | 0 | 0 | 6 |
|  |  | 5 | -29.62 | -53.69 | 102 | 13-Jun | 15-Jun | 20 | 100 | 4 | 0 | 0 | 0 | 4 |
|  |  | 6 | -29.62 | -53.69 | 102 | 13-Jun | 15-Jun | 20 | 100 | 10 | 0 | 0 | 0 | 10 |
|  | **MG** | 1 | -20.7 | -42.8 | 805 | 8-Jun | 10-Jun | 20 | 90 | 8 | 0 | 0 | 2 | 10 |
|  |  | 2 | -20.7 | -42.8 | 805 | 8-Jun | 10-Jun | 20 | 75 | 5 | 0 | 0 | 1 | 6 |
|  |  | 3 | -20.7 | -42.8 | 805 | 8-Jun | 10-Jun | 20 | 65 | 4 | 0 | 0 | 1 | 5 |
|  |  | 4 | -20.7 | -42.8 | 805 | 10-Jun | 12-Jun | 20 | 80 | 8 | 0 | 0 | 1 | 9 |
|  |  | 5 | -20.7 | -42.8 | 805 | 10-Jun | 12-Jun | 20 | 80 | 6 | 0 | 0 | 0 | 6 |
|  |  | 6 | -20.7 | -42.8 | 805 | 10-Jun | 12-Jun | 20 | 95 | 9 | 0 | 0 | 0 | 9 |
|  | **FD** | 1 | -16.03 | -47.77 | 864 | 15-May | 17-May | 12 | 100 | 2 | 0 | 0 | 0 | 2 |
|  |  | 2 | -16.03 | -47.77 | 864 | 5-Jun | 7-Jun | 12 | 91.67 | 3 | 0 | 0 | 0 | 3 |
|  |  | 3 | -15.61 | -48.08 | 1279 | 15-May | 17-May | 12 | 100 | 3 | 1 | 1 | 1 | 6 |
|  |  | 4 | -15.61 | -48.08 | 1279 | 5-Jun | 7-Jun | 12 | 100 | 5 | 0 | 0 | 0 | 5 |
|  |  | 5 | -15.66 | -48.11 | 1174 | 8-May | 10-May | 12 | 100 | 1 | 0 | 0 | 0 | 1 |
|  |  | 6 | -15.66 | -48.11 | 1174 | 29-May | 1-Jun | 12 | 100 | 2 | 0 | 0 | 0 | 2 |
|  |  | 7 | -15.99 | -47.85 | 1104 | 8-May | 10-May | 12 | 100 | 3 | 0 | 0 | 2 | 5 |
|  |  | 8 | -15.99 | -47.85 | 1104 | 29-May | 1-Jun | 12 | 100 | 1 | 0 | 0 | 0 | 1 |
|  |  | 9 | -15.56 | -48.03 | 1177 | 24-Apr | 28-Apr | 12 | 100 | 2 | 0 | 0 | 0 | 2 |
|  |  | 10 | -15.56 | -48.03 | 1177 | 22-May | 26-May | 12 | 100 | 2 | 0 | 0 | 0 | 2 |
|  |  | 11 | -15.65 | -48.2 | 1146 | 24-Apr | 28-Apr | 12 | 100 | 4 | 0 | 0 | 0 | 4 |
|  |  | 12 | -15.65 | -48.2 | 1146 | 22-May | 26-May | 12 | 100 | 3 | 0 | 0 | 0 | 3 |
|  | **PE** | 1 | -8.26 | -35.5 | 471 | 13-Jun | 15-May | 20 | 90 | 0 | 0 | 0 | 0 | 0 |
|  |  | 2 | -8.26 | -35.5 | 471 | 13-Jun | 15-May | 20 | 100 | 0 | 0 | 0 | 0 | 0 |
| **USA** | **TX** | 1 | 30.26 | -97.7 | 138 | 12-Sep | 14-Sep | 20 | 100 | 0 | 1 | 0 | 0 | 1 |
|  |  | 2 | 30.26 | -97.7 | 138 | 13-Sep | 15-Sep | 20 | 100 | 1 | 0 | 0 | 0 | 1 |
|  |  | 3 | 30.26 | -97.7 | 138 | 12-Sep | 14-Sep | 20 | 100 | 0 | 1 | 0 | 0 | 1 |
|  |  | 4 | 30.09 | -97.84 | 212 | 13-Sep | 15-Sep | 20 | 100 | 0 | 0 | 0 | 0 | 0 |
|  |  | 5 | 30.19 | -97.52 | 122 | 12-Sep | 14-Sep | 20 | 100 | 2 | 0 | 0 | 0 | 2 |
|  |  | 6 | 30.26 | -97.48 | 164 | 13-Sep | 15-Sep | 20 | 100 | 1 | 0 | 0 | 0 | 1 |
|  | **OK** | 1 | 35.5 | -97.53 | 364 | 6-Sep | 8-Sep | 20 | 100 | 1 | 1 | 0 | 0 | 2 |
|  |  | 2 | 35.5 | -97.53 | 364 | 6-Sep | 8-Sep | 20 | 100 | 1 | 0 | 0 | 0 | 1 |
|  |  | 3 | 35.67 | -97.5 | 357 | 6-Sep | 8-Sep | 20 | 100 | 5 | 0 | 0 | 0 | 5 |
|  |  | 4 | 35.56 | -97.47 | 349 | 6-Sep | 8-Sep | 20 | 100 | 2 | 0 | 0 | 0 | 2 |
|  | **KS** | 1 | 38.98 | -95.22 | 252 | 30-Aug | 1-Sep | 20 | 100 | 6 | 0 | 0 | 0 | 6 |
|  |  | 2 | 38.98 | -95.22 | 252 | 28-Aug | 30-Aug | 20 | 100 | 8 | 0 | 0 | 0 | 8 |
|  |  | 3 | 38.96 | -95.21 | 257 | 29-Aug | 31-Aug | 20 | 100 | 5 | 0 | 0 | 0 | 5 |
|  |  | 4 | 38.96 | -95.21 | 257 | 28-Aug | 30-Aug | 20 | 100 | 4 | 0 | 0 | 0 | 4 |
|  |  | 5 | 39.1 | -95.04 | 255 | 28-Aug | 30-Aug | 20 | 100 | 6 | 0 | 0 | 0 | 6 |
|  |  | 6 | 38.93 | -95.54 | 334 | 29-Aug | 31-Aug | 20 | 100 | 2 | 0 | 0 | 0 | 2 |
|  |  | 7 | 39.26 | -94.97 | 320 | 30-Aug | 1-Sep | 20 | 100 | 2 | 0 | 0 | 0 | 2 |
|  | **IA** | 1 | 41.77 | -92.72 | 307 | 22-Aug | 24-Aug | 20 | 100 | 6 | 2 | 0 | 0 | 8 |
|  |  | 2 | 41.76 | -92.71 | 310 | 22-Aug | 24-Aug | 20 | 100 | 6 | 0 | 0 | 0 | 6 |
|  |  | 3 | 41.16 | -93.2 | 296 | 23-Aug | 25-Aug | 20 | 100 | 7 | 1 | 0 | 0 | 8 |
|  |  | 4 | 41.48 | -95.05 | 391 | 23-Aug | 25-Aug | 20 | 100 | 5 | 1 | 0 | 0 | 6 |
|  |  | 5 | 41.76 | -93.81 | 294 | 21-Aug | 23-Aug | 20 | 95 | 7 | 0 | 0 | 0 | 7 |
|  |  | 6 | 41.67 | -93.74 | 272 | 22-Aug | 24-Aug | 20 | 95 | 7 | 0 | 0 | 0 | 7 |
|  | **MN** | 1 | 45 | -93.17 | 295 | 15-Aug | 17-Aug | 20 | 100 | 9 | 1 | 0 | 0 | 10 |
|  |  | 2 | 44.99 | -93.17 | 289 | 14-Aug | 16-Aug | 20 | 100 | 11 | 0 | 0 | 0 | 11 |
|  |  | 3 | 45.23 | -92.73 | 314 | 14-Aug | 16-Aug | 20 | 100 | 6 | 4 | 0 | 0 | 10 |
|  |  | 4 | 45.63 | -93.44 | 299 | 16-Aug | 18-Aug | 20 | 100 | 10 | 3 | 0 | 0 | 13 |
|  |  | 5 | 44.71 | -93.1 | 285 | 15-Aug | 17-Aug | 20 | 100 | 5 | 1 | 0 | 1 | 7 |
|  |  | 6 | 45.07 | -93.85 | 284 | 15-Aug | 17-Aug | 20 | 100 | 10 | 0 | 0 | 0 | 10 |
|  |  |  |  |  |  |  | **totals** | **1004** | **97** | **249** | **17** | **1** | **9** | **276** |


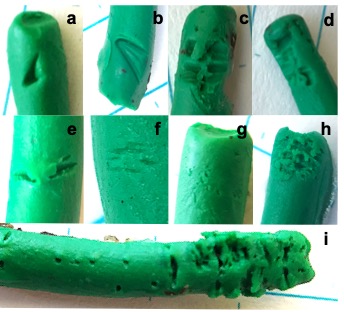


**Supplementary Fig. S1.** Example of bite marks of birds (a, b), mammals (c, d), arthropods (e-i) on model caterpillar baits.

**Supplementary Table S2.** Model summaries for generalized linear mixed-effect models of binomial predation on artificial caterpillar baits by arthropod, bird, mammal, and all predators with site nested within country as a random effect. Pearson’s χ2 goodness of fit tests, R^2^_GLMM_ values, model estimates and Wald statistics reported at the 95% confidence interval.

| **ARTHROPOD** |  |  |  |  |  |  |  |  |
| --- | --- | --- | --- | --- | --- | --- | --- | --- |
| Pearson’s *χ*^2^_(49)_ = 33.90, *p* = 0.950; R^2^_Marginal_ = 0.75, R^2^_Conditional_ = 0.75 | | | | | | | | |
| **Fixed effect** | **Parameter** | **Est.** | **SE** | **95% CI** | | **Wald χ^2^** | ***z* value** | ***p*** |
| intercept | USA-intercept | -6.31 | 1.50 | -9.25 | -3.36 | 37.56 | -1.14 | <0.001 |
| **latitude** |  | **0.07** | **0.02** | **0.03** | **0.11** | **10.36** | **-0.47** | **0.001** |
| country | Brazil-intercept | -3.05 | 0.50 | -4.03 | -2.08 | 17.63 | -1.17 | <0.001 |
| **latitude:country** |  | **0.12** | **0.04** | **0.04** | **0.20** | **8.65** | **-1.10** | **0.003** |
|  | **USA-slope** | **0.19** | **0.03** | **0.12** | **0.26** | **30.09** | **5.49** | **<0.001** |
|  | **Brazil-slope** | **0.07** | **0.02** | **0.03** | **0.11** | **10.36** | **3.22** | **0.001** |
|  |  |  |  |  |  |  |  |  |
| **BIRD** |  |  |  |  |  |  |  |  |
| Pearson’s *χ*^2^_(26)_ = 20.23, p = 0.078; R^2^_M_ = 0.83, R^2^_C_ = 0.83 | | | | | | | | |
| **Fixed effect** | **Parameter** | **Est.** | **SE** | **95% CI** | | **Wald χ^2^** | ***z* value** | ***p*** |
| intercept | USA-intercept | -11.10 | 3.99 | -18.92 | -3.28 | 7.74 | -2.78 | 0.005 |
| latitude | USA-slope | 0.17 | 0.10 | -0.02 | 0.36 | 3.19 | 1.79 | 0.074 |
|  |  |  |  |  |  |  |  |  |
| **MAMMAL** |  |  |  |  |  |  |  |  |
| Pearson’s *χ*^2^_(23)_ = 17.31, p = 0.793; R^2^_M_ = 0.17, R^2^_C_ = 0.17 | | | | | | | | |
| **Fixed effect** | **Parameter** | **Est.** | **SE** | **95% CI** | | **Wald χ^2^** | ***z* value** | ***p*** |
| intercept | intercept | -3.94 | 1.49 | -6.86 | -1.02 | 6.99 | -2.64 | 0.008 |
| latitude | latitude | -0.03 | 0.07 | -0.18 | 0.11 | 0.19 | -0.43 | 0.665 |
|  |  |  |  |  |  |  |  |  |
| **ALL PREDATORS** |  |  |  |  |  |  |  |  |
| Pearson’s *χ*^2^_(49)_ = 36.81, p = 0.900; R^2^_M_ = 0.77, R^2^_C_ = 0.77 | | | | | | | | |
| **Fixed effect** | **Parameter** | **Est.** | **SE** | **95% CI** | | **Wald χ^2^** | ***z* value** | ***p*** |
| intercept | USA-intercept | -7.11 | 1.47 | -9.99 | -4.23 | 23.42 | -4.84 | <0.001 |
| **latitude** |  | **0.06** | **0.02** | **0.02** | **0.10** | **8.47** | **2.91** | **0.004** |
| country | Brazil-intercept | -2.72 | 0.47 | -3.64 | -1.80 | 33.64 | -5.80 | <0.001 |
| **latitude:country** |  | **0.14** | **0.04** | **0.07** | **0.22** | **13.39** | **3.66** | **<0.001** |
|  | **USA-slope** | **0.20** | **0.03** | **0.14** | **0.27** | **36.69** | **6.06** | **<0.001** |
|  | **Brazil-slope** | **0.06** | **0.02** | **0.02** | **0.10** | **8.47** | **2.91** | **0.004** |

**Supplementary Table S3.** Plant densities per 80 pins identified by with point quadrat method. Pins were arranged in two perpendicular 20 m rows of 40 vertical pins every half meter for each plot by site. *indicates species flowering at time of survey.

|  |  | **plot number** | | | | | | | | | | | |
| --- | --- | --- | --- | --- | --- | --- | --- | --- | --- | --- | --- | --- | --- |
| **family** | **species** | **1** | **2** | **3** | **4** | **5** | **6** | **7** | **8** | **9** | **10** | **11** | **12** |
| **Pernambuco (PE), Brazil** | |  |  |  |  |  |  |  |  |  |  |  |  |
| Amaranthaceae | unknown | 0.021 | 0.103 |  |  |  |  |  |  |  |  |  |  |
| Asteraceae | *Ageratum conyzoides** | 0.043 | 0.103 |  |  |  |  |  |  |  |  |  |  |
|  | *Galinsoga parviflora** | 0.191 | 0.256 |  |  |  |  |  |  |  |  |  |  |
| Commelinaceae | *Commelina difusa** | 0.043 | 0.077 |  |  |  |  |  |  |  |  |  |  |
| Cyperaceae | *Cyperus esculentus* | 0.170 | 0.077 |  |  |  |  |  |  |  |  |  |  |
| Poaceae | *Echinochloacrus galli* | 0.234 | 0.231 |  |  |  |  |  |  |  |  |  |  |
|  | *Eleusine indica* | 0.021 | 0.026 |  |  |  |  |  |  |  |  |  |  |
|  | **bare ground** | 0.277 | 0.128 |  |  |  |  |  |  |  |  |  |  |
| **Minas Gerais (MG), Brazil** | |  |  |  |  |  |  |  |  |  |  |  |  |
| Amaryllidaceae | unknown |  |  | no data | 0.030 |  |  |  |  |  |  |  |  |
| Asteraceae | *Ageratum conyzoides** | present | 0.230 |  | 0.080 |  |  |  |  |  |  |  |  |
| Brassicaceae | *Brassica juncea** |  |  |  | 0.030 | 0.070 |  |  |  |  |  |  |  |
|  | *Brassica oleraceae* | present | 0.560 |  | 0.600 | 0.460 | 0.450 |  |  |  |  |  |  |
| Caryophyllaceae | *Stellaria media* | present |  |  |  |  |  |  |  |  |  |  |  |
| Cyperaceae | *Cyperus esculentus* |  |  |  |  | 0.010 |  |  |  |  |  |  |  |
|  | *Cyperus rotundus* | present | 0.080 |  | 0.160 | 0.090 | 0.010 |  |  |  |  |  |  |
| Fabaceae | *Trifolium** |  |  |  |  |  | 0.130 |  |  |  |  |  |  |
| Lamiaceae | *Rosmarinus officinalis* |  |  |  |  | 0.020 | 0.020 |  |  |  |  |  |  |
| Musaceae | *Musa acuminata* |  |  |  | 0.050 |  |  |  |  |  |  |  |  |
| Poaceae | *Cyperus rotundus* |  |  |  |  | 0.250 |  |  |  |  |  |  |  |
|  | *Zea mays* |  |  |  |  | 0.030 |  |  |  |  |  |  |  |
|  | **bare ground** | present | 0.140 |  | 0.060 | 0.070 | 0.380 |  |  |  |  |  |  |
| **District Federal (DF), Brazil** | |  |  |  |  |  |  |  |  |  |  |  |  |
| Amaranthaceae | *Amaranthus* sp. | 0.034 | 0.020 | 0.027 | 0.100 | 0.014 | 0.045 | 0.042 |  |  |  | 0.089 | 0.021 |
|  |  | 0.034 | 0.020 | 0.027 | 0.100 |  | 0.045 | 0.042 |  |  |  | 0.089 | 0.021 |
|  | *Amaranthus viridis* |  |  |  |  | 0.014 |  |  |  |  |  |  |  |
| *Amaryllidaceae* | *Allium* sp. |  |  |  |  |  |  |  |  | 0.025 | 0.028 |  |  |
|  | *Allium sativum* |  |  | 0.123 |  |  |  |  |  | 0.192 |  |  |  |
| Asteraceae | *Ageratum conyzoides** |  |  | 0.109 |  | 0.010 | 0.005 |  |  | 0.010 |  |  |  |
|  | *Bidens alba** | 0.148 | 0.087 |  | 0.128 | 0.005 | 0.015 | 0.169 | 0.024 |  |  | 0.037 | 0.051 |
|  | *Emilia sonchifolia** |  |  |  |  |  |  | 0.069 |  | 0.005 |  |  |  |
|  | *Galinsoga ciliate** |  |  | 0.041 |  |  |  |  |  |  |  |  |  |
|  | *Galinsoga parviflora** |  |  |  |  | 0.005 | 0.010 |  |  |  |  | 0.042 | 0.031 |
|  | *Taraxacum* sp.* |  |  |  |  | 0.005 |  |  |  |  |  | 0.005 | 0.021 |
|  | *Tithonia diversifolia** |  | 0.005 |  |  |  |  |  |  | 0.030 |  |  |  |
|  | unknown |  |  |  |  |  |  |  |  | 0.005 |  |  |  |
| Brassicaceae | *Brassica oleracea* | 0.199 | 0.163 | 0.245 | 0.283 | 0.308 | 0.515 | 0.092 | 0.265 | 0.233 | 0.116 | 0.707 | 0.646 |
| Cannaceae | *Canna** |  |  |  |  |  |  | 0.042 |  |  |  |  |  |
| Caricaceae | *Carica papaya* |  |  |  |  |  |  |  |  | 0.020 | 0.005 |  |  |
| Commelinaceae | *Commelina difusa** | 0.114 | 0.056 | 0.050 | 0.041 |  | 0.025 | 0.131 | 0.035 | 0.020 | 0.005 |  |  |
| Convolvulaceae | *Ipomoea purpurea* | 0.205 | 0.306 | 0.055 | 0.119 |  |  | 0.165 | 0.106 |  |  |  |  |
|  | *Merremia* sp. |  |  |  |  | 0.005 |  |  |  |  |  |  |  |
|  | unknown |  |  | 0.009 |  |  |  |  |  |  |  |  |  |
| Cucurbitaceae | *Cucumis anguria* |  |  | 0.009 |  |  |  |  |  |  |  |  |  |
| Cyperaceae | *Cyperus rotundus* |  |  | 0.077 | 0.114 |  |  |  |  | 0.015 |  |  |  |
| Dioscoreaceae | *Dioscorea* sp. |  |  |  |  |  |  |  |  | 0.030 | 0.051 |  |  |
| Euphorbiaceae | *Euphorbia heterophylla* |  |  |  |  |  |  |  |  |  |  |  | 0.010 |
|  | *Manihot esculenta* |  |  |  |  |  |  |  |  | 0.005 | 0.037 |  |  |
|  | *Ricinus communis* L.* |  |  |  |  |  |  |  |  | 0.005 |  |  |  |
| Fabaceae | *Leguminosae* sp. |  |  |  |  |  |  |  |  | 0.020 |  |  |  |
|  | *Trifolium* sp.* | 0.131 |  | 0.032 |  | 0.038 | 0.070 |  |  |  |  |  | 0.015 |
| Malvaceae | *Sida rhombifolia** |  |  |  |  |  |  |  |  | 0.005 |  |  |  |
| Musaceae | *Musa acuminata* |  |  |  |  |  |  |  |  | 0.005 |  |  |  |
| Poaceae | *Cyperus rotundus* | 0.006 |  |  |  |  |  | 0.200 |  |  |  |  | 0.010 |
| Portulacaceae | *Portulaca oleracea* |  |  |  |  |  |  |  |  |  |  | 0.005 |  |
| Rubiaceae | *Coffea arabica** |  |  |  |  |  |  |  |  | 0.114 | 0.060 |  |  |
| Solanaceae | *Solanum lycopersicum** | 0.006 |  |  |  |  |  |  |  |  |  |  |  |
|  | **bare ground** | 0.159 | 0.362 | 0.223 | 0.215 | 0.611 | 0.315 | 0.088 | 0.571 | 0.262 | 0.698 | 0.115 | 0.195 |
| **Rio Grande do Sul (RS), Brazil** | |  |  |  |  |  |  |  |  |  |  |  |  |
| Asteraceae | *Erigeron bonariensis** |  |  |  | 0.012 |  |  |  |  |  |  |  |  |
|  | *Galinsoga ciliate** | 0.013 |  |  |  |  |  |  |  |  |  |  |  |
|  | *Gnaphalium spicatum* |  |  |  | 0.136 | 0.100 | 0.088 |  |  |  |  |  |  |
|  | *Soliva sessilis* |  |  |  | 0.012 |  |  |  |  |  |  |  |  |
|  | *Sonchus oleraceus* |  |  |  |  |  | 0.013 |  |  |  |  |  |  |
|  | unknown |  | 0.013 | 0.038 |  |  |  |  |  |  |  |  |  |
| Brassicaceae | *Brassica oleracea* | 0.675 | 0.600 | 0.550 | 0.296 | 0.288 | 0.363 |  |  |  |  |  |  |
| Caryophyllaceae | *Sperguula arvensis* | 0.075 | 0.013 | 0.025 |  |  |  |  |  |  |  |  |  |
|  | *Stellaria media* |  |  |  |  | 0.025 | 0.050 |  |  |  |  |  |  |
| Commelinaceae | *Commelina benghalensis** |  |  |  | 0.074 |  |  |  |  |  |  |  |  |
| Convolvulaceae | *Ipomoea batatas* |  |  |  | 0.049 | 0.013 | 0.025 |  |  |  |  |  |  |
| Cyperaceae | *Cyperus esculentus* |  |  |  | 0.049 | 0.025 |  |  |  |  |  |  |  |
| Malvaceae | *Sida cordifolia** |  |  |  |  | 0.013 |  |  |  |  |  |  |  |
|  | *Sida corniculate** |  |  |  | 0.025 |  |  |  |  |  |  |  |  |
| Oxalidaceae | *Oxalis corniculate** |  |  |  | 0.074 | 0.213 | 0.100 |  |  |  |  |  |  |
| Poaceae | *Eleusine indica* | 0.050 | 0.050 | 0.025 |  |  |  |  |  |  |  |  |  |
|  | unknown |  |  |  | 0.049 | 0.088 | 0.088 |  |  |  |  |  |  |
| Polygonaceae | *Persicaria maculosa* |  |  |  |  | 0.013 |  |  |  |  |  |  |  |
| Rubiaceae | *Richardia brasiliensis** |  |  |  | 0.012 |  | 0.050 |  |  |  |  |  |  |
|  | **bare ground** | 0.188 | 0.325 | 0.363 | 0.210 | 0.225 | 0.225 |  |  |  |  |  |  |
| **Texas (TX), United States** | |  |  |  |  |  |  |  |  |  |  |  |  |
| Amaranthaceae | *Amaranthus palmeri** |  |  | 0.100 |  |  |  |  |  |  |  |  |  |
|  | *Chenopodium berlandieri* |  |  |  | 0.025 |  |  |  |  |  |  |  |  |
| Asteraceae | *Calendula officinalis** |  |  |  | 0.038 |  |  |  |  |  |  |  |  |
| Brassicaceae | *Brassica oleracea* | 0.038 | 0.200 | 0.213 | 0.418 | 0.342 | 0.088 |  |  |  |  |  |  |
|  | *Brassica rapa* | 0.025 |  |  |  |  |  |  |  |  |  |  |  |
| Fabaceae | *Trifolium* sp.*** |  |  |  |  |  | 0.013 |  |  |  |  |  |  |
| Poaceae | *Digitaria* sp. | 0.025 | 0.100 | 0.063 | 0.025 | 0.013 | 0.050 |  |  |  |  |  |  |
| Portulacaceae | *Portulaca oleracea* |  |  | 0.063 | 0.025 |  |  |  |  |  |  |  |  |
|  | **bare ground** | 0.913 | 0.700 | 0.563 | 0.468 | 0.646 | 0.850 |  |  |  |  |  |  |
| **Oklahoma (OK), United States** | |  |  |  |  |  |  |  |  |  |  |  |  |
| Apiaceae | *Daucus carota* | 0.013 |  |  |  |  |  |  |  |  |  |  |  |
| Brassicaceae | *Brassica oleracea* | 0.288 | 0.075 | 0.038 | 0.038 |  |  |  |  |  |  |  |  |
|  | *Brassica rapa* |  |  |  | 0.125 |  |  |  |  |  |  |  |  |
| Convolvulaceae | *Convolvulus arvensis** |  | 0.025 |  |  |  |  |  |  |  |  |  |  |
|  | *Ipomoea batatas* |  |  | 0.125 |  |  |  |  |  |  |  |  |  |
| Cucurbitaceae | *Cucurbita* sp. |  | 0.188 |  |  |  |  |  |  |  |  |  |  |
| Fabaceae | *Phaseolus vulgaris* | 0.088 |  |  |  |  |  |  |  |  |  |  |  |
| Malvaceae | *Abelmoschus esculentus** | 0.313 |  |  | 0.050 |  |  |  |  |  |  |  |  |
| Poaceae | *Setaria faberi* | 0.025 |  |  |  |  |  |  |  |  |  |  |  |
|  | unknown |  | 0.150 |  |  |  |  |  |  |  |  |  |  |
| Solanaceae | *Capsicum annuum** |  | 0.213 |  |  |  |  |  |  |  |  |  |  |
|  | **bare ground** | 0.275 | 0.350 | 0.838 | 0.788 |  |  |  |  |  |  |  |  |
| **Kansas (KS), United States** | |  |  |  |  |  |  |  |  |  |  |  |  |
| Amaranthaceae | *Amaranthus palmeri** |  |  | 0.100 |  |  | 0.115 | 0.272 |  |  |  |  |  |
|  | *Chenopodium berlandieri** |  |  |  |  |  | 0.038 |  |  |  |  |  |  |
| Amaryllidaceae | *Allium cepa* |  |  |  |  | 0.074 |  |  |  |  |  |  |  |
| Apiaceae | *Foeniculum vulgare** |  |  |  |  | 0.037 |  |  |  |  |  |  |  |
| Asteraceae | *Calendula officinalis** |  |  |  |  |  | 0.192 |  |  |  |  |  |  |
| Brassicaceae | *Brassica oleracea* | 0.363 | 0.139 | 0.238 | 0.308 |  | 0.064 | 0.321 |  |  |  |  |  |
|  | *Brassica rapa* |  |  |  |  | 0.222 |  |  |  |  |  |  |  |
|  | *Eruca sativa* |  |  |  |  |  | 0.064 |  |  |  |  |  |  |
| Convolvulaceae | *Ipomoea purpurea* |  | 0.051 |  |  |  |  |  |  |  |  |  |  |
|  | *Euphorbiaceae* sp. |  |  |  |  |  | 0.051 |  |  |  |  |  |  |
|  | *Euphorbia corollate** |  |  |  |  |  | 0.051 |  |  |  |  |  |  |
| Lamiaceae | *Ocimum basilicum** |  |  |  |  | 0.049 |  |  |  |  |  |  |  |
| Poaceae | *Digitaria* | 0.300 |  | 0.038 | 0.026 | 0.025 |  |  |  |  |  |  |  |
|  | *Digitaria sanguinalis* | 0.338 | 0.519 | 0.450 | 0.167 |  | 0.103 |  |  |  |  |  |  |
|  | *Setaria faberi* |  | 0.177 | 0.050 | 0.013 |  |  | 0.099 |  |  |  |  |  |
|  | *Setaria pumila* |  | 0.051 |  |  |  |  |  |  |  |  |  |  |
|  | *Zea mays* |  |  | 0.063 |  |  |  |  |  |  |  |  |  |
| Portulacaceae | *Portulaca oleracea* |  |  |  |  |  | 0.090 |  |  |  |  |  |  |
| Rosaceae | *Rubus idaeus* |  |  |  |  | 0.012 |  |  |  |  |  |  |  |
| Solanaceae | *Solanum carolinese** |  | 0.063 |  |  |  |  | 0.086 |  |  |  |  |  |
|  | *Solanum lycopersicum** |  |  |  |  | 0.025 |  |  |  |  |  |  |  |
|  | **bare ground** |  |  | 0.063 | 0.487 | 0.556 | 0.282 | 0.222 |  |  |  |  |  |
| **Iowa (IA), United States** | |  |  |  |  |  |  |  |  |  |  |  |  |
| Amaranthaceae | *Amaranthus palmeri** | 0.169 | 0.012 | 0.013 |  | 0.355 |  |  |  |  |  |  |  |
|  | *Chenopodium berlandieri** | 0.039 | 0.025 |  |  |  | 0.037 |  |  |  |  |  |  |
| Amaryllidaceae | *Allium ampeloprasum* |  |  | 0.053 |  |  |  |  |  |  |  |  |  |
| Brassicaceae | *Brassica oleracea* | 0.636 | 0.889 | 0.213 | 0.838 | 0.105 | 0.506 |  |  |  |  |  |  |
| Caryophyllaceae | *Stellaria media* |  |  |  |  |  | 0.049 |  |  |  |  |  |  |
| Cucurbitaceae | *Cucurbita* |  |  | 0.160 |  |  |  |  |  |  |  |  |  |
| Fabaceae | *Phaseolus vulgaris* |  |  | 0.040 |  |  |  |  |  |  |  |  |  |
|  | *Trifolium sp.** |  | 0.025 |  |  |  |  |  |  |  |  |  |  |
| Malvaceae | *Abutilon theophrasti* |  |  | 0.027 |  |  | 0.012 |  |  |  |  |  |  |
| Poaceae | *Digitaria* |  |  | 0.067 |  |  | 0.086 |  |  |  |  |  |  |
|  | *Setaria faberi* |  |  |  |  | 0.539 |  |  |  |  |  |  |  |
| Polygonaceae | *Rumex* sp. |  | 0.025 |  |  |  |  |  |  |  |  |  |  |
| Portulacaceae | *Portulaca oleracea* |  |  |  |  |  | 0.198 |  |  |  |  |  |  |
|  | **bare ground** | 0.156 | 0.025 | 0.427 | 0.163 |  | 0.111 |  |  |  |  |  |  |
| **Minnesota (MN), United States** | |  |  |  |  |  |  |  |  |  |  |  |  |
| Amaranthaceae | *Amaranthus palmeri** | 0.063 | 0.012 | 0.088 | 0.449 | 0.075 | 0.026 |  |  |  |  |  |  |
|  | *Chenopodium berlandieri** | 0.213 |  | 0.075 | 0.192 |  | 0.103 |  |  |  |  |  |  |
| Apiaceae | *Daucus carota* |  |  |  |  |  | 0.013 |  |  |  |  |  |  |
| Brassicaceae | *Brassica oleracea* | 0.350 | 0.317 | 0.588 | 0.141 | 0.688 | 0.359 |  |  |  |  |  |  |
|  | *Brassica rapa* |  | 0.110 |  |  |  |  |  |  |  |  |  |  |
| Caryophyllaceae | *Stellaria media* |  |  |  |  |  | 0.013 |  |  |  |  |  |  |
| Fabaceae | *Vicia villosa* |  |  |  |  |  | 0.026 |  |  |  |  |  |  |
| Malvaceae | *Abutilon theophrasti* |  | 0.037 |  |  |  |  |  |  |  |  |  |  |
| Poaceae | *Digitaria sp.* | 0.213 | 0.024 | 0.013 | 0.077 | 0.025 |  |  |  |  |  |  |  |
|  | *Digitaria sanguinalis* |  |  |  |  |  | 0.141 |  |  |  |  |  |  |
|  | *Setaria faberi* |  |  |  |  |  | 0.256 |  |  |  |  |  |  |
| Portulacaceae | *Portulaca oleracea* | 0.100 | 0.415 | 0.013 |  | 0.013 |  |  |  |  |  |  |  |
| Solanaceae | *Solanum carolinese** | 0.038 | 0.049 |  |  |  |  |  |  |  |  |  |  |
|  | **bare ground** | 0.025 | 0.037 | 0.225 | 0.141 | 0.200 | 0.064 |  |  |  |  |  |  |

**Supplementary Table S4.** Model summaries for generalized linear mixed-effect model of binomial predation on artificial caterpillar baits by all predators testing whether selecting for a range of plant diversity within a locality has the potential to obscure latitudinal patterns, with site nested within country as a random effect. Pearson’s χ^2^ goodness of fit tests, R^2^_GLMM_ values, model estimates and Wald statistics reported at the 95% confidence interval.

| Pearson’s χ^2^_(34)_ = 21.35, *p* = 0.955; R^2^_M_ = 0.81, R^2^_C_ = 0.81 | | | | |
| --- | --- | --- | --- | --- |
| **Fixed effect** | **Wald χ^2^** | **df** | ***p*** |  |
| intercept | 9.15 | 1.00 | 0.002 |  |
| plant richness | 0.05 | 1.00 | 0.822 |  |
| site | 2.38 | 7.00 | 0.936 |  |
| plant richness:site | 2.77 | 7.00 | 0.906 |  |

**Supplementary Table S5.** Model summaries for generalized linear mixed-effect model of binomial predation on artificial caterpillar baits by arthropod with site nested within country as a random effect. Full model of all interactions of scaled latitude, elevation, and Köppen aridity index (higher values = less arid), as well as country and selected model based on likelihood ratio tests of fixed effect removal reported. Pearson’s χ2 goodness of fit tests, R^2^_GLMM_ values, and Wald statistics reported at the 95% confidence interval.

| **FULL MODEL** |  |  |  |  |  |
| --- | --- | --- | --- | --- | --- |
| Pearson’s χ^2^_(37)_ = 17.75, *p* = 1.000; R^2^_M_ = 1.00, R^2^_C_ = 1.00 | | | | | |
| **Fixed effect** | **Wald χ2** | **df** | ***p*** |  |  |
| Intercept | 1.23 | 1 | 0.266 |  |  |
| latitude | 1.11 | 1 | 0.292 |  |  |
| country | 2.13 | 1 | 0.144 |  |  |
| elevation | 0.45 | 1 | 0.503 |  |  |
| AI | 0.61 | 1 | 0.435 |  |  |
| latitude:country | 0.42 | 1 | 0.516 |  |  |
| latitude:elevation | 0.35 | 1 | 0.555 |  |  |
| country:elevation | 0.05 | 1 | 0.815 |  |  |
| latitude:AI | 0.32 | 1 | 0.575 |  |  |
| country:AI | 0.19 | 1 | 0.660 |  |  |
| elevation:AI | 0.84 | 1 | 0.359 |  |  |
| latitude:country:elevation | 1.12 | 1 | 0.289 |  |  |
| latitude:country:AI | 0.53 | 1 | 0.466 |  |  |
| latitude:elevation:AI | 0.77 | 1 | 0.380 |  |  |
| country:elevation:AI | 3.11 | 1 | 0.078 |  |  |
| latitude:country:elevation:AI | 0.04 | 1 | 0.845 |  |  |
|  |  |  |  |  |  |
| **SELECTED MODEL** | |  |  |  |  |
| Pearson’s χ^2^_(46)_ = 24.80, *p* = 1.00; R^2^_M_ = 0.90, R^2^_C_ = 0.90 | | | | |  |
| **Fixed effect** | **Wald χ2** | **df** | ***p*** |  |  |
| Intercept | 0.70 | 1 | 0.402 |  |  |
| latitude | 0.00 | 1 | 0.954 |  |  |
| country | 1.52 | 1 | 0.218 |  |  |
| AI | 0.38 | 1 | 0.537 |  |  |
| **country:AI** | **12.98** | **1** | **<0.001** |  |  |
| latitude:AI | 3.32 | 1 | 0.068 |  |  |

**Supplementary Table S6.** Model summary for generalized linear mixed-effect model of the effect of Köppen aridity index (AI, higher values = less arid) by country on binomial predation on artificial caterpillar baits by arthropod with site nested within country as a random effect. Pearson’s χ2 goodness of fit tests, R^2^_GLMM_ values, estimates and Wald statistics reported at the 95% confidence interval.

| **ARTHROPOD** |  |  |  |  |  |  |  |  |
| --- | --- | --- | --- | --- | --- | --- | --- | --- |
| Pearson’s χ^2^_(49)_ = 28.56, *p* = 0.991; R^2^_M_ = 0.46, R^2^_C_ = 0.76 | | | | | | | | |
| **Fixed effect** | **Parameter** | **Estimate** | **SE** | **95% CI** | | **Wald χ^2^** | ***z* value** | ***p*** |
| intercept | USA-intercept | -1.19 | 2.64 | -6.36 | 3.98 | 0.20 | -0.45 | 0.653 |
| **AI** |  | **0.15** | **0.07** | **0.02** | **0.28** | **5.11** | **2.26** | **0.024** |
| **country** | **Brazil-intercept** | **-5.39** | **1.68** | **-8.68** | **-2.10** | **10.32** | **-3.21** | **0.001** |
| AI:country |  | 0.09 | 0.12 | -0.15 | 0.33 | 0.53 | 0.73 | 0.467 |

**Supplementary Table S7.** Arthropod predator counts surveyed within 0.5m of 12 focal *Brassica* plants in each plot.

|  |  | **plot number** | | | | | | | | | | | |
| --- | --- | --- | --- | --- | --- | --- | --- | --- | --- | --- | --- | --- | --- |
|  | **family** | **1** | **2** | **3** | **4** | **5** | **6** | **7** | **8** | **9** | **10** | **11** | **12** |
| **Pernambuco (PE), Brazil** | |  |  |  |  |  |  |  |  |  |  |  |  |
|  | Araneae | 2 | 1 |  |  |  |  |  |  |  |  |  |  |
|  | Coccinellidae |  | 1 |  |  |  |  |  |  |  |  |  |  |
| **Minas Gerais (MG), Brazil** | |  |  |  |  |  |  |  |  |  |  |  |  |
|  | Araneae | 3 | 1 | 1 | 2 |  | 1 |  |  |  |  |  |  |
|  | Carabidae |  | 1 | 1 |  | 1 |  |  |  |  |  |  |  |
|  | Syrphidae (larvae) |  |  | 1 |  |  |  |  |  |  |  |  |  |
|  | Vespidae |  |  | 1 |  |  |  |  |  |  |  |  |  |
| **District Federal (DF), Brazil** | |  |  |  |  |  |  |  |  |  |  |  |  |
|  | Araneae | 7.20 | 5.06 | 4.49 | 4.00 | 6.02 | 2.04 | 4.52 | 1.48 | 8.03 | 10.50 | 1.02 | 3.00 |
|  | Asilidae | 2.45 |  |  |  |  |  |  |  |  |  |  | 0.49 |
|  | Carabidae |  | 0.52 |  |  |  | 2.00 |  | 0.50 |  |  |  | 0.00 |
|  | Coccinellidae | 42.31 |  |  |  |  | 1.02 |  |  |  | 10.96 | 15.51 | 0.50 |
|  | Dolichopodidae | 3.01 |  | 0.50 | 2.03 | 1.51 | 1.03 | 0.49 | 0.51 |  | 2.05 |  |  |
|  | Forficulidae |  |  |  |  |  |  |  |  |  | 0.49 |  |  |
|  | Neuroptera (larvae) | 12.25 | 14.91 |  | 0.48 | 0.51 | 1.47 |  | 1.50 | 2.02 | 4.50 | 5.55 |  |
|  | Staphylinidae |  |  |  |  |  |  |  |  | 7.64 |  |  |  |
|  | Syrphidae (larvae) | 0.52 |  | 0.49 | 2.01 | 2.58 | 0.49 | 1.02 |  |  | 3.01 | 1.98 | 2.01 |
|  | Vespidae |  |  | 3.39 |  |  |  |  | 0.49 |  |  |  | 0.48 |
| **Rio Grande do Sul (RS), Brazil** | |  |  |  |  |  |  |  |  |  |  |  |  |
|  | Araneae |  |  | 1 | 2 | 2 | 6 |  |  |  |  |  |  |
|  | Forficulidae |  |  |  | 2 | 2 |  |  |  |  |  |  |  |
|  | Reduviidae |  |  |  |  |  | 1 |  |  |  |  |  |  |
| **Texas (TX), United States** | |  |  |  |  |  |  |  |  |  |  |  |  |
|  | Araneae |  | 1 | 5 | 4 | 2 | 1 |  |  |  |  |  |  |
|  | Carabidae |  |  |  |  | 3 |  |  |  |  |  |  |  |
|  | Neuroptera (larvae) |  |  | 10 | 1 | 1 |  |  |  |  |  |  |  |
| **Oklahoma (OK), United States** | |  |  |  |  |  |  |  |  |  |  |  |  |
|  | Araneae |  |  | 1 | 4 |  |  |  |  |  |  |  |  |
|  | Forficulidae | 1 |  |  |  |  |  |  |  |  |  |  |  |
| **Kansas (KS), United States** | |  |  |  |  |  |  |  |  |  |  |  |  |
|  | Anthocoridae |  |  |  |  | 8 |  |  |  |  |  |  |  |
|  | Araneae | 1 | 2 |  | 3 |  | 3 | 3 |  |  |  |  |  |
|  | Carabidae | 2 |  |  |  |  |  |  |  |  |  |  |  |
|  | Coccinellidae | 3 |  |  | 1 |  |  |  |  |  |  |  |  |
|  | Lampyridae (larvae) |  |  |  |  | 2 |  |  |  |  |  |  |  |
|  | Neuroptera (larvae) |  |  | 1 |  |  |  |  |  |  |  |  |  |
| **Iowa (IA), United States** | |  |  |  |  |  |  |  |  |  |  |  |  |
|  | Anthocoridae |  |  |  |  |  | 2 |  |  |  |  |  |  |
|  | Araneae |  | 6 | 4 | 0 | 1 |  |  |  |  |  |  |  |
|  | Coccinellidae |  |  | 35 |  | 21 | 11 |  |  |  |  |  |  |
|  | Meloidae (larvae) |  |  |  |  | 4 |  |  |  |  |  |  |  |
|  | Neuroptera |  | 16 | 4 |  |  | 8 |  |  |  |  |  |  |
|  | Syrphidae (larvae) | 1 |  |  |  | 2 |  |  |  |  |  |  |  |
|  | Vespidae |  |  |  |  |  | 2 |  |  |  |  |  |  |
| **Minnesota (MN), United States** | |  |  |  |  |  |  |  |  |  |  |  |  |
|  | Anthocoridae |  |  |  | 1 |  |  |  |  |  |  |  |  |
|  | Araneae | 3 | 1 | 1 |  |  | 3 |  |  |  |  |  |  |
|  | Carabidae |  |  | 1 |  |  |  |  |  |  |  |  |  |
|  | Coccinellidae | 46 | 63 |  | 4 | 6 | 1 |  |  |  |  |  |  |
|  | Neuroptera (larvae) |  | 17 |  | 2 |  |  |  |  |  |  |  |  |
|  | Opiliones |  |  | 2 |  |  |  |  |  |  |  |  |  |

**Supplementary Table S8.** Invertebrate prey counts surveyed within 0.5m of 12 focal *Brassica* plants in each plot.

|  |  | **plot number** | | | | | | | | | | | |
| --- | --- | --- | --- | --- | --- | --- | --- | --- | --- | --- | --- | --- | --- |
|  | **taxa** | **1** | **2** | **3** | **4** | **5** | **6** | **7** | **8** | **9** | **10** | **11** | **12** |
| **Pernambuco (PE), Brazil** | |  |  |  |  |  |  |  |  |  |  |  |  |
|  | Chrysomelidae |  | 1 |  |  |  |  |  |  |  |  |  |  |
|  | Lepidoptera (larvae) | 8 | 7 |  |  |  |  |  |  |  |  |  |  |
|  | Lypaphis erysime | 2 | 2 |  |  |  |  |  |  |  |  |  |  |
|  | Veronicelloidea | 1 |  |  |  |  |  |  |  |  |  |  |  |
| **Minas Gerais (MG), Brazil** | |  |  |  |  |  |  |  |  |  |  |  |  |
|  | Acrididae | 1 |  |  |  | 1 |  |  |  |  |  |  |  |
|  | Aleyrodidae |  |  | 1 | 5 | 3 |  |  |  |  |  |  |  |
|  | Chrysomelidae |  | 1 |  |  |  |  |  |  |  |  |  |  |
|  | Drosophilidae |  |  | 17 |  |  |  |  |  |  |  |  |  |
|  | Lepidoptera (larvae) |  |  | 2 | 1 | 3 | 1 |  |  |  |  |  |  |
|  | Myzus persicae | 151 | 18 | 44 | 272 | 139 | 30 |  |  |  |  |  |  |
|  | Thripidae |  |  |  | 1 |  |  |  |  |  |  |  |  |
|  | Veronicelloidea |  |  |  |  | 1 | 1 |  |  |  |  |  |  |
| **District Federal (DF), Brazil** | |  |  |  |  |  |  |  |  |  |  |  |  |
|  | Acrididae | 0.51 |  |  |  | 0.48 |  |  |  |  | 0.50 |  |  |
|  | Agromyzidae |  |  |  |  |  |  | 0.49 |  |  |  |  |  |
|  | Aleyrodidae |  | 2.51 | 0.52 |  | 2.50 | 1.50 | 0.98 | 1.02 | 20.15 |  | 1.03 | 1.42 |
|  | Brevicoryne brassicae | 3786.77 | 33.57 | 16.58 | 103.36 | 273.12 | 452.08 | 60.36 |  | 483.22 | 1641.99 | 325.30 | 895.77 |
|  | Chrysomelidae | 3.03 | 13.17 |  | 0.48 | 2.49 |  | 1.01 |  |  | 1.00 |  | 1.47 |
|  | Cicadellidae | 0.99 |  |  |  | 0.48 | 0.50 |  |  | 0.53 |  |  |  |
|  | Gryllidae |  |  | 0.51 |  |  |  |  |  |  |  |  |  |
|  | Lepidoptera (larvae) | 27.52 | 10.08 | 2.54 | 22.31 | 4.06 | 1.00 |  | 3.01 | 80.52 | 4.99 | 1.48 | 0.51 |
|  | Lygaeidae |  |  |  |  |  |  |  |  |  | 0.00 |  |  |
|  | Miridae | 1.00 |  |  |  |  |  | 0.99 | 0.50 |  |  |  | 0.50 |
|  | Myzus persicae | 129.61 |  | 2.51 | 29.28 | 59.85 |  | 18.98 | 46.08 | 209.71 | 1682.40 | 13.61 | 2.50 |
|  | Pentatomidae | 1.02 | 1.01 |  |  |  | 0.49 |  |  |  |  |  |  |
|  | Tettigoniidae |  |  |  |  |  |  | 0.96 |  | 0.46 |  |  |  |
|  | Thripidae |  |  |  |  |  | 2.97 |  |  |  |  | 1.02 | 7.86 |
|  | Tingidae |  |  | 0.50 |  |  |  |  |  |  |  |  |  |
|  | Veronicelloidea | 77.84 |  | 7.50 |  | 4.52 | 0.99 |  | 1.03 | 120.19 | 61.19 |  |  |
| **Rio Grande do Sul (RS), Brazil** | |  |  |  |  |  |  |  |  |  |  |  |  |
|  | Agromyzidae | 2 | 5 | 6 | 1 |  |  |  |  |  |  |  |  |
|  | Aleyrodidae | 22 | 13 | 19 | 7 | 4 | 4 |  |  |  |  |  |  |
|  | Chrysomelidae | 2 | 4 | 1 | 4 | 7 | 4 |  |  |  |  |  |  |
|  | Gryllidae | 1 |  |  |  |  | 1 |  |  |  |  |  |  |
|  | Lepidoptera (larvae) | 5 | 2 | 1 | 10 | 6 | 2 |  |  |  |  |  |  |
|  | Meloidae |  | 1 |  |  |  |  |  |  |  |  |  |  |
|  | Sciaridae | 4 | 4 |  |  |  |  |  |  |  |  |  |  |
|  | Veronicelloidea |  |  |  | 4 | 6 | 18 |  |  |  |  |  |  |
| **Texas (TX), United States** | |  |  |  |  |  |  |  |  |  |  |  |  |
|  | Acrididae |  |  |  |  | 1 |  |  |  |  |  |  |  |
|  | Agromyzidae |  | 6 |  |  |  | 3 |  |  |  |  |  |  |
|  | Aleyrodidae | 44 | 91 | 36 | 5 | 8 | 3 |  |  |  |  |  |  |
|  | Brevicoryne brassicae | 13 | 28 | 22 |  |  |  |  |  |  |  |  |  |
|  | Lepidoptera (larvae) | 1 | 5 | 34 | 46 | 15 | 56 |  |  |  |  |  |  |
| **Oklahoma (OK), United States** | |  |  |  |  |  |  |  |  |  |  |  |  |
|  | Aleyrodidae |  | 2 | 3 | 14 |  |  |  |  |  |  |  |  |
|  | Brevicoryne brassicae | 6 |  |  |  |  |  |  |  |  |  |  |  |
|  | Chrysomelidae |  |  | 1 |  |  |  |  |  |  |  |  |  |
|  | Lepidoptera (larvae) |  | 41 | 15 | 18 |  |  |  |  |  |  |  |  |
|  | Myzus persicae |  | 8 | 2 | 7 |  |  |  |  |  |  |  |  |
|  | Pentatomidae | 2 |  |  |  |  |  |  |  |  |  |  |  |
| **Kansas (KS), United States** | |  |  |  |  |  |  |  |  |  |  |  |  |
|  | Acrididae |  |  | 3 | 1 |  |  |  |  |  |  |  |  |
|  | Aleyrodidae |  |  |  |  |  | 7 | 3 |  |  |  |  |  |
|  | Aphis nerii |  |  |  |  |  |  | 12 |  |  |  |  |  |
|  | Brevicoryne brassicae |  |  |  |  | 270 | 59 |  |  |  |  |  |  |
|  | Chrysomelidae | 1 |  | 1 |  | 66 |  |  |  |  |  |  |  |
|  | Cicadellidae | 1 |  |  |  |  |  |  |  |  |  |  |  |
|  | Lepidoptera (larvae) | 2 |  | 45 | 6 | 30 | 58 | 16 |  |  |  |  |  |
|  | Macrosiphum euphorbiae |  |  |  |  | 14 | 1 |  |  |  |  |  |  |
|  | Miridae |  |  |  |  | 1 |  |  |  |  |  |  |  |
|  | Pentatomidae | 4 | 113 | 79 | 39 | 3 |  | 1 |  |  |  |  |  |
|  | Tingidae |  |  |  |  |  | 1 |  |  |  |  |  |  |
| **Iowa (IA), United States** | |  |  |  |  |  |  |  |  |  |  |  |  |
|  | Aleyrodidae | 25 | 10 |  |  |  | 2 |  |  |  |  |  |  |
|  | Chrysomelidae | 1 |  | 6 | 4 | 1 | 9 |  |  |  |  |  |  |
|  | Cicadellidae | 1 |  |  |  |  |  |  |  |  |  |  |  |
|  | Drosophilidae |  |  |  |  |  | 1 |  |  |  |  |  |  |
|  | Lepidoptera (larvae) | 10 | 4 |  | 44 | 5 | 10 |  |  |  |  |  |  |
|  | Macrosiphum euphorbiae |  | 1 |  |  |  |  |  |  |  |  |  |  |
|  | Myzus persicae | 19 | 530 | 1737 | 10 | 65 | 118 |  |  |  |  |  |  |
|  | Pentatomidae |  |  |  |  | 2 |  |  |  |  |  |  |  |
|  | Thripidae |  |  |  |  |  | 4 |  |  |  |  |  |  |
| **Minnesota (MN), United States** | |  |  |  |  |  |  |  |  |  |  |  |  |
|  | Aleyrodidae | 1 |  |  |  |  |  |  |  |  |  |  |  |
|  | Lepidoptera (larvae) | 10 | 29 | 42 | 57 | 28 | 29 |  |  |  |  |  |  |
|  | Miridae |  |  |  |  |  | 1 |  |  |  |  |  |  |
|  | Myzus persicae | 1 | 4 | 46 | 39 | 87 | 17 |  |  |  |  |  |  |
|  | Pentatomidae |  |  | 0 |  |  |  |  |  |  |  |  |  |
|  | Thripidae |  |  | 3 |  |  |  |  |  |  |  |  |  |

**Supplementary Table S9.** PiecewiseSEM model of factors influencing daily arthropod predator attack rates (predation) with site nested within country as a random effect. (Fisher’s C = 25.16, *df* = 24, *p* = 0.397).

| Response | Predictor | Estimate | SE | *df* | Crit. value | *p* |
| --- | --- | --- | --- | --- | --- | --- |
| predation | **aridity** | **0.58** | **0.20** | **7.61** | **8.31** | **0.022** |
|  | predator richness | -0.02 | 0.13 | 46.18 | 0.03 | 0.857 |
|  | predator density | 0.10 | 0.11 | 43.05 | 0.92 | 0.344 |
|  | **plant density** | **0.22** | **0.09** | **41.49** | **5.20** | **0.028** |
|  | plant richness | -0.15 | 0.11 | 44.17 | 1.97 | 0.167 |
| aridity | **latitude** | **1.49** | **0.29** | **7.75** | **23.34** | **0.001** |
| predator richnness | aridity | -0.02 | 0.18 | 0.04 | 0.00 | 0.995 |
|  | latitude | -0.13 | 0.19 | 0.01 | 0.01 | 0.995 |
|  | plant density | 0.06 | 0.11 | 41.80 | 0.28 | 0.602 |
|  | plant richness | -0.05 | 0.13 | 43.03 | 0.11 | 0.737 |
|  | prey richness | 0.13 | 0.16 | 45.95 | 0.65 | 0.426 |
|  | **prey density** | **0.38** | **0.11** | **41.94** | **10.96** | **0.002** |
| predator density | plant density | 0.13 | 0.11 | 44.65 | 1.15 | 0.290 |
|  | plant richness | 0.14 | 0.14 | 44.81 | 0.90 | 0.349 |
|  | prey richness | -0.19 | 0.15 | 39.87 | 1.23 | 0.275 |
|  | **prey density** | **0.48** | **0.13** | **41.05** | **13.02** | **0.001** |
|  | **predator richness** | **0.33** | **0.14** | **37.65** | **4.31** | **0.045** |
|  | aridity | 0.15 | 0.14 | 8.03 | 0.82 | 0.391 |
| prey richness | latitude | -0.01 | 0.29 | 2.42 | 0.00 | 0.987 |
|  | plant richness | 0.21 | 0.11 | 47.23 | 3.35 | 0.073 |
| prey density | plant density | 0.00 | 0.14 | 49.51 | 0.00 | 0.981 |
| plant richness | **plant density** | **0.32** | **0.12** | **48.91** | **6.90** | **0.012** |
|  | latitude | -0.15 | 0.23 | 5.09 | 0.16 | 0.707 |
